# Our New York Letter

**Published:** 1890-02

**Authors:** 


### OUR NEW YORK LETTER.

NEW YORK, January 12, 1890.

The question of operation in peritonitis was the subject under consideration at the last meeting of the Academy. A paper was read by Dr. W. Gill Wylie. He believed that the majority of cases, of both local and general peritonitis, could be cured by surgical measures. So-called chronic pelvic cellulitis, which was really a local peritonitis, if resulting in the formation of pus, could be treated radically and certainly only by laparotomy. He believed abscesses about the cæcum to be intra-peritoneal, and that a free and safe outlet for the pus should always be made. The peritonitis in such cases was usually local, but if the openings from the bowels were large, it would be general. Sometimes a tumor could be discovered only after the patient was anæsthetized, and sometimes not even then. Pain in the iliac region, with tenderness on pressure, and chilly sensations might be the only signs present. When general peritonitis was present, but the seat of disease could not be located, an incision should be made in the median line. If an abscess were then discovered which could be opened more advantageously by a lateral incision, that in the median line could be immediately closed. If the peritonitis were found to be due to an already ruptured abscess, or to have been general from the beginning, a large drainage tube should be inserted and the cavity washed out with water at 110° or 115° F. In all cases the incision should be large enough to allow of the introduction of the hand, and all adhesions should be broken up. This was a very important point. It was usual for the edges of the wound to slough in such cases, owing to the fetid character of the pus. It was better to operate early that rupture of the abscess might be avoided. An exploratory incision did not increase the risk and might save the patient's life. The histories of five cases of typhlitis and peri-typhlitis, which had been successfully operated on, were cited in detail to illustrate these points.

Dr. Wm. M. Polk regarded the chances of a successful operation in general peritonitis as very slight, especially in cases of a septic character after labor or abortion. Cleansing of the peirtoneal cavity in peritonitis after laparotomy was usually of no avail. He thought it was an excellent thing to break up adhesions, but the intestines were usually distended with gas, and the necessary manipulations caused them to protrude so much that an increase of shock was the result. Packing the wound with iodoform gauze, leaving an end out for drainage, he regarded as a more thorough method of cleansing than a drainage-tube. There were two general classes of local peritonitis, that from perforation of some portion of the intestinal tract, and that from diseases of the uterus or its appendages. Cases of the first class were all dangerous, and demanded early operation to anticipate trouble. It could not be known at the beginning whether any case would recover spontaneously, would result in abscess, or would terminate in general peritonitis and death. The operation to be considered was simply incision. If this were done, general peritonitis would be prevented; the cutting necessary to open an abscess would only be done a little earlier, and the chances of recovery after an early operation were greater than that the case would result in spontaneous recovery. He concluded then, that in all cases of local peritonitis of the first class it was best to make an early incision. In cases resulting from affections of the uterus or its appendages, however, the case was different. Those due to gonorrhœa or to traumatism within the uterus usually resulted in abscess and required incision. In other cases such an operation was seldom desirable, except when general peritonitis seemed possible or pus was suspected. In the majority of cases, however, the operation only added another burden to the patient.

Dr. A. G. Gerster thought that no certain formulæ regarding operation could be laid down until more observations of the natural history of these conditions had been made. The cases operated on by gynecologists were, as a rule, chronic and not dangerous to life. The general surgeon was called upon to operate on cases due to lesions in the intestinal tract, which were usually acute, and accompanied with great depression. He approved of Dr. Wylie's plan of making an exploratory incision in the median line when the exact source of the peritonitis was uncertain. He had seen a case illustrating the advantage of this not long before, in which he found an abscess of the kidney, which he readily opened from behind, after closing the median wound immediately

In closing the discussion, Dr. Wylie remarked that death after laparotomy was oftener due to intestinal obstruction than to peritonitis. Hence the necessity of breaking up adhesions. Symptoms of the formation of pus were always indications for an operation. There were some post-partum cases suitable for operation, but he had not intended to include them in his statement.

A discussion of the subject of empyema in children occupied the last meeting of the section on Pædiatrics. Dr. R. Abbé introduced the subject. He regarded suppurative joint troubles and empyema as analogous affections. Some cases of both got well whether operated on or not. In such cases the effusion was not pure pus, but largely serous. If pure, yellow pus were present its escape was a *sine qua non* to satisfactory recovery, Aspiration could not be recommended, as the cavity could not then be washed out. Incision and drainage was the method which appealed to every surgeon. The strictest antisepsis, even to the use of the spray, was necessary during the operation. A close-fitting dressing to prevent the entrance of air during inspiration, while not preventing the escape of the pus, promoted expansion of the lung. More than four-fifths of non-tubercular cases so treated recovered.

Dr. F. Huber agreed with Dr. Abbé that the success of aspiration in some cases was because of the sero-purulent rather than the purely purulent character of the fluid in these cases.

When much pus and fibrous flocculi were present, a free incision should be made. Thirty out of thirty-five cases treated by him had recovered. No permanent sinuses were left, nor had resection of the ribs been found necessary. The cavity should be irrigated once after operation to remove large lymph masses. He did not regard the spray as necessary.

Dr. Gerster said that when the masses of lymph were large some cases baffled even incision, unless it were properly made. It was usually too far forward and not low enough. It ought to be back of the axillary line and as low as between the eighth and ninth ribs. In this way the dome of the diaphragm was exposed, and formed an inclined plane which facilitated drainage. This was an important point. He did not think strict antisepsis necessary in these cases. Only one thorough irrigation should be made. When the lung was shrunk and surrounded by a dense membrane, the cavity sometimes failed to close. In such a case resection of several ribs sometimes succeeded. He had removed portions of as many as nine ribs, and even part of the clavicle. This was a very bloody operation, however, and could not be performed on a weak patient. A Russian surgeon had recently suggested merely making a section of the ribs at two points, corresponding to the edges of the cavity to be obliterated, and the pressing of the portions of ribs thus separated into the cavity by means of dressings. This was a much less bloody operation.

Dr. W. P. Northrup remarked that he had frequently noticed at autopsies on empyema cases, that the lung was well expanded, and it was only after pushing it aside that the pus in the pleural cavity was discovered. It had occurred to him that it was easy in such cases to make the incision too far forward. It should be as far back as the axillary line at least. The fifth, sixth or seventh intercostal space might be chosen in different cases. The sixth allowed of the breaking up of adhesions with a bent probe. The operation should be done early, as a rule. The removal of a portion of a rib was a good plan, or a metal tube could be placed between the ribs. Aspiration before incision was a useful measure in promoting expansion of the lung. He had made some experiments on a dog, which showed that expansion of the lung was promoted by a valvular canula, which allowed the pus to escape but prevented the entrances of air to the pleural cavity during inspiration.

Dr. A. L. Loomis said that he had observed cases of empyema before aspirators were invented, when they were treated by aspiration, and when they were treated by free incision and drainage. A very large proportion of cases began as an acute affection, complicating pneumonia. Such cases recovered under any plan of treatment. He did not regard pleurisy complicating pneumonia in children as very serious. He believed then that if a case assumed a chronic character, or if the fluid accumulated rapidly and respiration was interfered with, a free incision should be made ; not otherwise. Subacute cases, due to tuberculosis, were nearly always fatal. Aspiration or incision relieved the symptoms, but hastened the progress of the disease.

The incision should be posterior to the axillary line, and low down. Resection of a rib was often necessary to prevent a suppurating sinus, gradual wasting, waxy changes, and death.

Dr. J. E. Winters stated that his rule was to make a free incision as soon as pus was discovered. Recovery was hastened by this treatment, in all cases that would recover. The incision should be at the lowest possible point. This could be determined by careful location of the bottom of the thoracic cavity, on the sound side. An aspirating needle attached to a hypodermic syringe should then be introduced to make sure that the chest cavity would be reached; and the needle should be used as a guide for the incision. The finger should be used if necessary to remove masses of fibrin. The majority of cases recovered without resection of a rib.

Dr. Gerster added that he had seen the diagnosis of serous effusion into the pleural cavity made because the needle of the asperator had been introduced in front, and at too high a point. An opening lower down, posteriorly, revealed pus.

